# Supplementary material for: Syngas as Electron Donor for Sulfate and Thiosulfate Reducing Haloalkaliphilic Microorganisms in a Gas-Lift Bioreactor
Source: Microorganisms. 2020 Sep 22;8(9):1451. doi: 10.3390/microorganisms8091451 (PMC7565546; doi:10.3390/microorganisms8091451)
Supplement: Supplementary file 1 [file microorganisms-08-01451-s001.pdf]

Supplementary Files

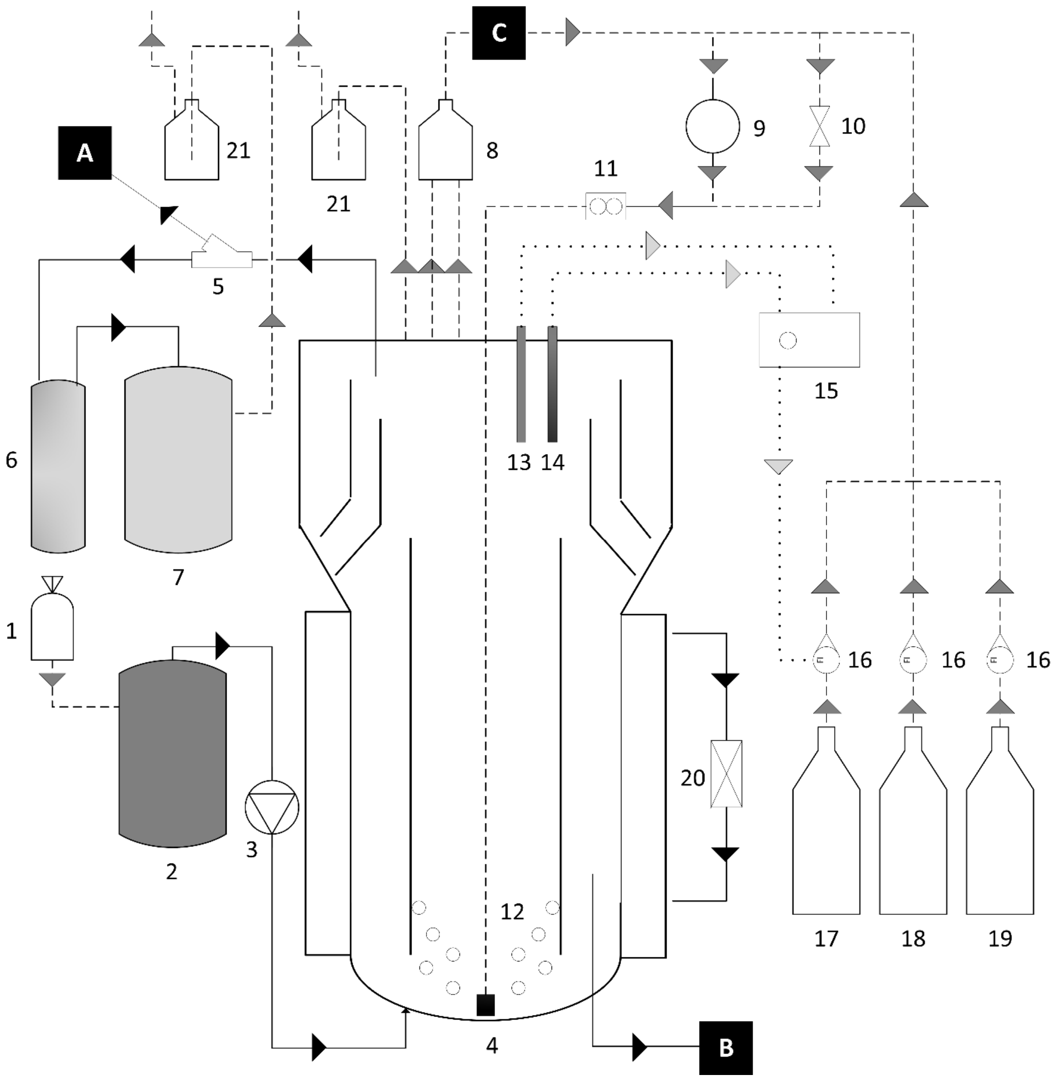

Supplementary Figure 1 - Scheme of the gas-lift bioreactor set-up. A – outlet sampling point; B – biomass sampling point; C – gas sampling point; 1 – N_2 gas bag; 2 – medium bottle; 3 – membrane pump; 4 – gas-lift bioreactor with 3 phase separator; 5 – sampling valve; 6 – long water lock; 7 – effluent bottle; 8 – condenser; 9 – vacuum pump; 10 – gas flow adjusting valve; 11 – gas flow meter; 12 – diffuser; 13 – ORP sensor; 14 – pH sensor; 15 – controller; 16 – mass flow controller; 17 – CO2 gas bottle; 18 – H2 gas bottle; 19 – CO bottle; 20 – thermostat bath; 21 – water lock.

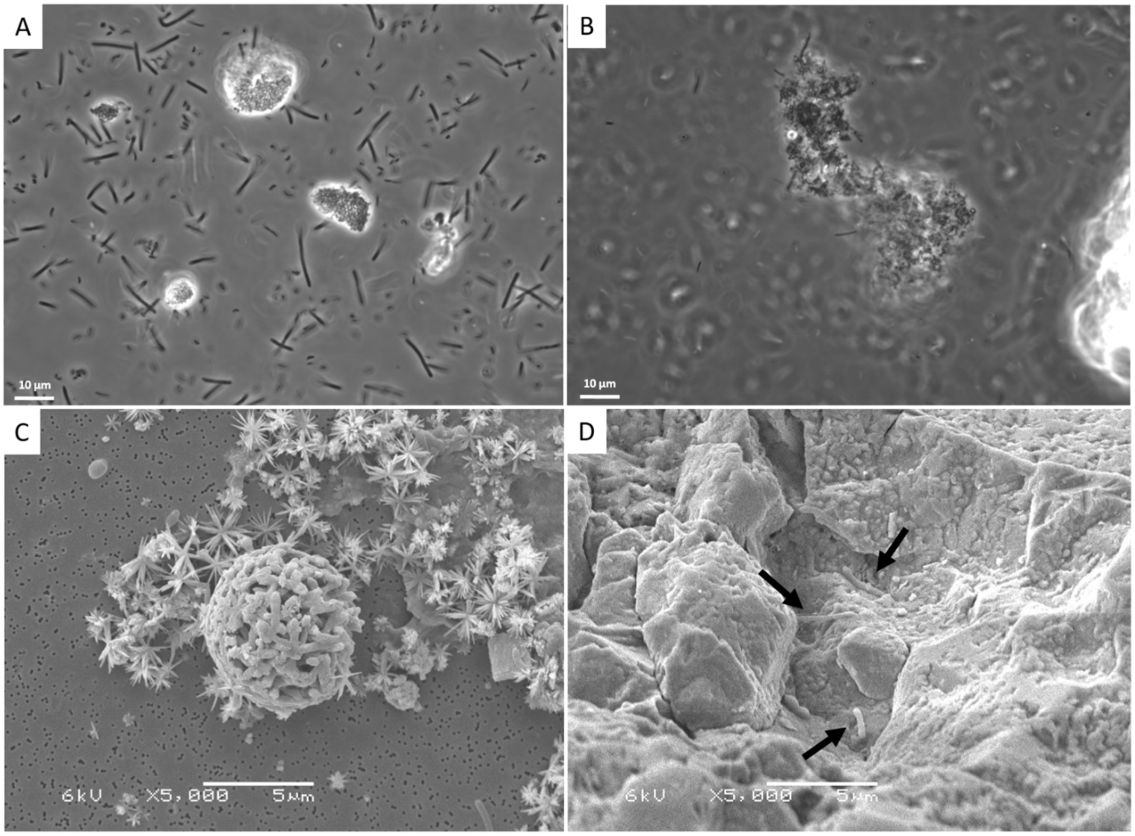

Supplementary Figure 2 – Microscopic observations of the biomass from the bioreactor. A – Small compact aggregates observed on day 154 and representative for the whole period with 15% CO (scale bar represents 10 µm). B – Small aggregates observed on a previous bioreactor operated with H_2 as electron donor (Sousa et al., 2020); (scale bar represents 10 µm). C – Scanning electron micrograph of a small aggregate from a sample from day 78 (scale bar represents 5 µm). D – Scanning electron micrograph of microorganisms attached to sand inside cavities on a sample from day 78 (scale bar represents 5 µm; black arrows point to microorganisms).
